# A second monoclinic polymorph of 2,3-di­phenyl­pyrazine

**DOI:** 10.1107/S2056989026000460

**Published:** 2026-01-23

**Authors:** Abdulkarim K. Albishri, Naser E. Eltayeb, Jamal Lasri, Tuncer Hökelek, Aidan P. McKay

**Affiliations:** a Department of Chemistry College of Science and Arts King Abdulaziz University, Rabigh 21911 Saudi Arabia; b Department of Chemistry Rabigh College of Science and Arts King Abdulaziz University,Jeddah 21589 Saudi Arabia; c Department of Chemistry, Faculty of Pure and Applied Sciences, International University of Africa, Khartoum 2469, Sudan; d Department of Physics, Hacettepe University, 06800 Beytepe, Ankara, Türkiye; e EaStCHEM School of Chemistry, University of St Andrews, Fife, KY16 9ST, United Kingdom; University of Aberdeen, United Kingdom

**Keywords:** 2,3-di­phenyl­pyrazine, crystal structure, hy­dro­gen bonding, π-stacking, Hirshfeld surface

## Abstract

The title com­pound, C_16_H_12_N_2_ (**I**), crystallizes in the space group *P*2_1_/*c* with one mol­ecule in the asymmetric unit, in which the dihedral angles between the planes of the pyrazine ring and pendant phenyl rings are 53.12 (3) and 33.28 (3)°. In the crystal, pairwise C—H⋯N hy­dro­gen bonds link the mol­ecules into centrosymmetric dimers and aromatic π–π stacking inter­actions between the pyrazine rings of adjacent mol­ecules and C—H⋯π inter­actions help to consolidatate the packing. Compound **I** is a polymorph of the previously reported form of 2,3-di­phenyl­pyrazine [Kitano *et al.* (1983 ▸). *Acta Cryst.* C**39**, 136–139], which crystallizes in the space group *C*2/*c* with two mol­ecules in the asymmetric unit. The Hirshfeld surfaces and energy frameworks of the two polymorphs are com­pared and the bonding modes of the mol­ecules as ligands are surveyed.

## Abstract 2

In a second monoclinic polymorph of 2,3-di­phenyl­pyrazine, pairwise C—H⋯N hy­dro­gen bonds link the mol­ecules into centrosymmetric dimers and aromatic π–π stacking inter­actions between the pyrazine rings of adjacent mol­ecules and C—H⋯π inter­actions help to consolidatate the packing.

## 1. Chemical context

The title com­pound **I** belongs to the class of organic com­pounds known as pyrazines or 1,4-diazines (Mason, 1887 ▸; Ohta *et al.*, 1982 ▸). Pyrazine derivatives are of inter­est due to their pharmaceutical activities and natural occurence (Sammes, 1975 ▸; Cheeseman & Werstiuk, 1972 ▸). The syntheses and reactivities of pyrazine analogues have been investigated by Akita & Ohta (1982 ▸). Currently, our research focuses on the syntheses, reactivities and anti­cancer activities of a variety of cyclic and acyclic imine (C=N)-type com­pounds (*e.g.* Eltayeb *et al.*, 2025 ▸) and the crystal structure determination of **I** was undertaken as part of these studies. Compound **I** is a polymorph of the previously reported form of 2,3-di­phenyl­pyrazine (Kitano *et al.*, 1983 ▸) with Cambridge Structural Database (CSD; Groom *et al.*, 2016 ▸) refcode BOHPOD in the space group *C*2/*c* with two mol­ecules in the asymmetric unit.
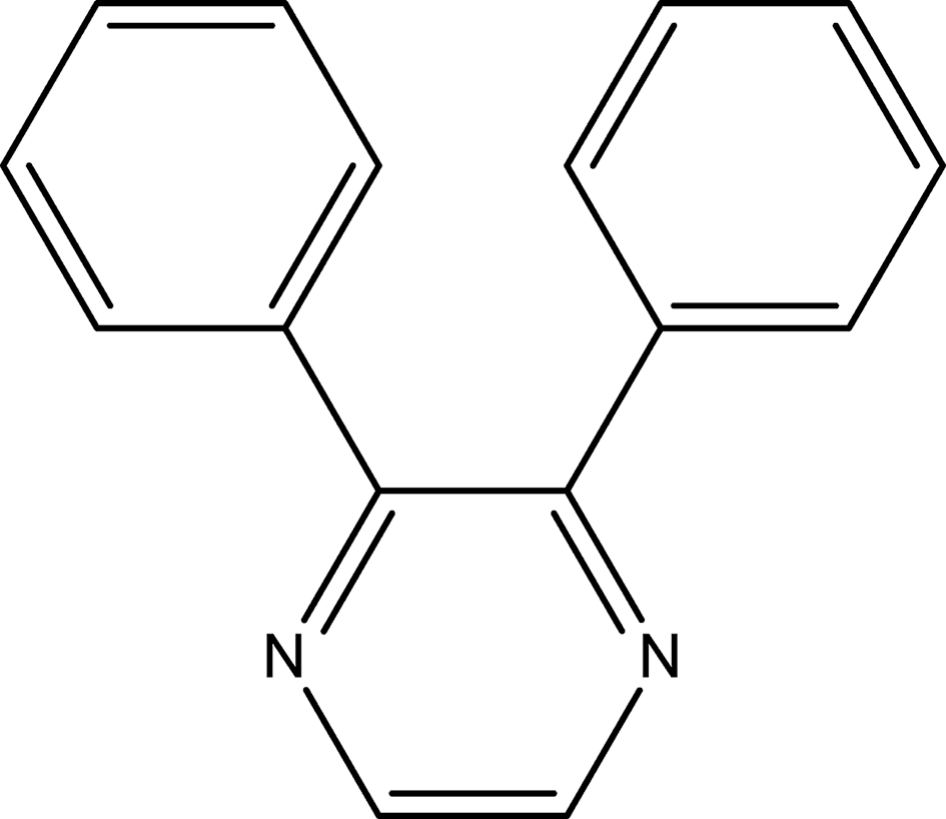


## 2. Structural commentary

**Figure 1:**
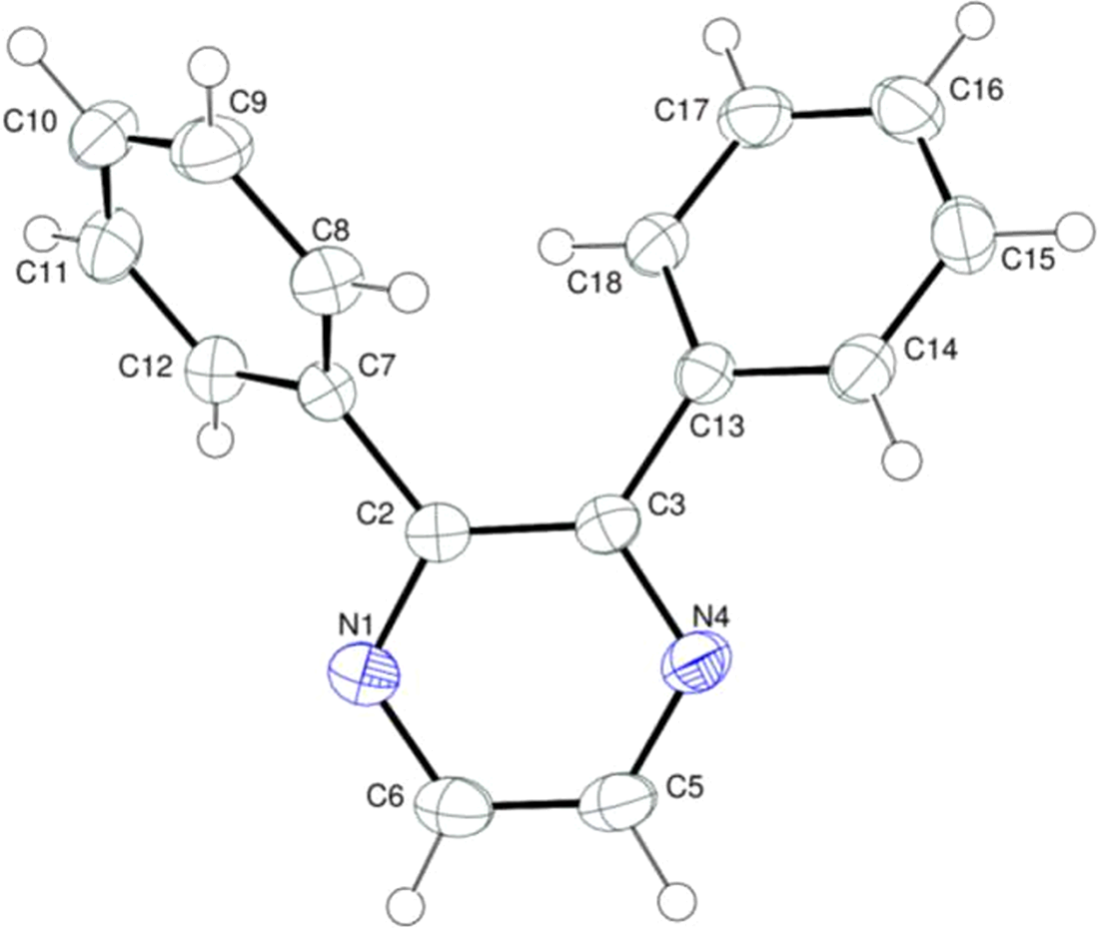
The mol­ecular structure of **I**, showing displacement ellipsoids at the 50% probability level.

Compound **I** crystallizes in the space group *P*2_1_/*c* with one mol­ecule in the asymmetric unit (Fig. 1 ▸). It contains a pyrazine ring, *A* (N1/N4/C2–C6), and pendant phenyl rings, *B* (C7–C12) and *C* (C13–C18), oriented at dihedral angles of *A*/*B* = 53.12 (3)°, *A*/*C* = 33.28 (3)° and *B*/*C* = 64.72 (3)°. The *B* and *C* rings are rotated in the same sense from the *A* ring plane due to steric hinderence between them and the C7—C2—C3—C13 torsion angle is −12.78 (16)°. The pyrazine ring in **I** is distinctly twisted, with an r.m.s. deviation of 0.043 Å for the six atoms and C6—N1—C2—C3 and C6—C5—N4—C3 torsion angles of 4.82 (15) and 2.75 (16)°, respectively.

## 3. Supra­molecular features

**Figure 2:**
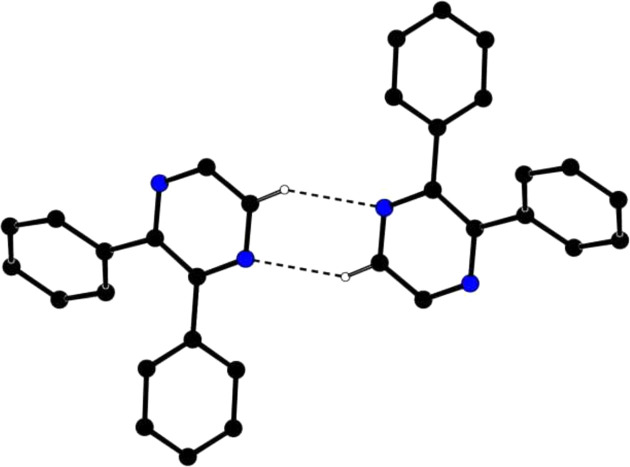
A partial packing diagram of **I**, showing an inversion dimer linked by pairwise C—H⋯N hy­dro­gen bonds (dashed lines). The other H atoms have been omitted for clarity.

In the crystal of **I**, pairwise C6—H6⋯N1^i^ hy­dro­gen bonds (Table 1 ▸) link the mol­ecules into centrosymmetric dimers (Fig. 2 ▸) enclosing 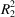
(6) loops. Aromatic π–π stacking inter­actions between the pyrazine rings of adjacent mol­ecules, with an inter-centroid distance of 3.5711 (6) Å (slippage = 1.213 Å), and C—H⋯π inter­actions (Table 1 ▸) help to consolidate the crystal packing. In polymorph BOPHOD, only directional C—H⋯π inter­actions are present and the densities of **I** and BOPHOD of 1.278 and 1.233 Mg m^−3^, respectively, suggest that **I** is the more stable.

## 4. Hirshfeld surface analysis

**Figure 3:**
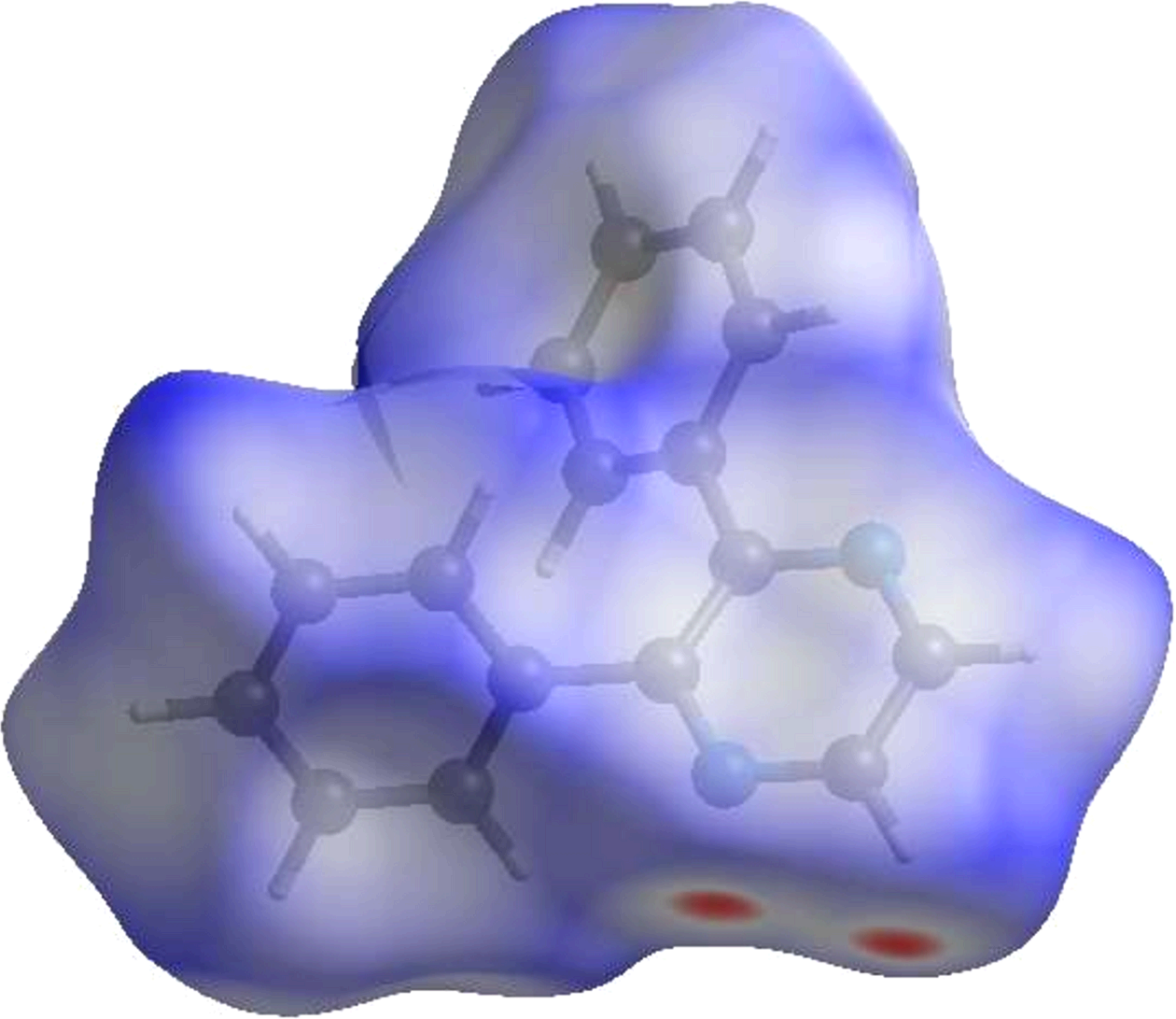
The three-dimensional Hirshfeld surface of **I** plotted over *d*_norm_ in the range from −0.17 to 1.34 a.u.

**Figure 4:**
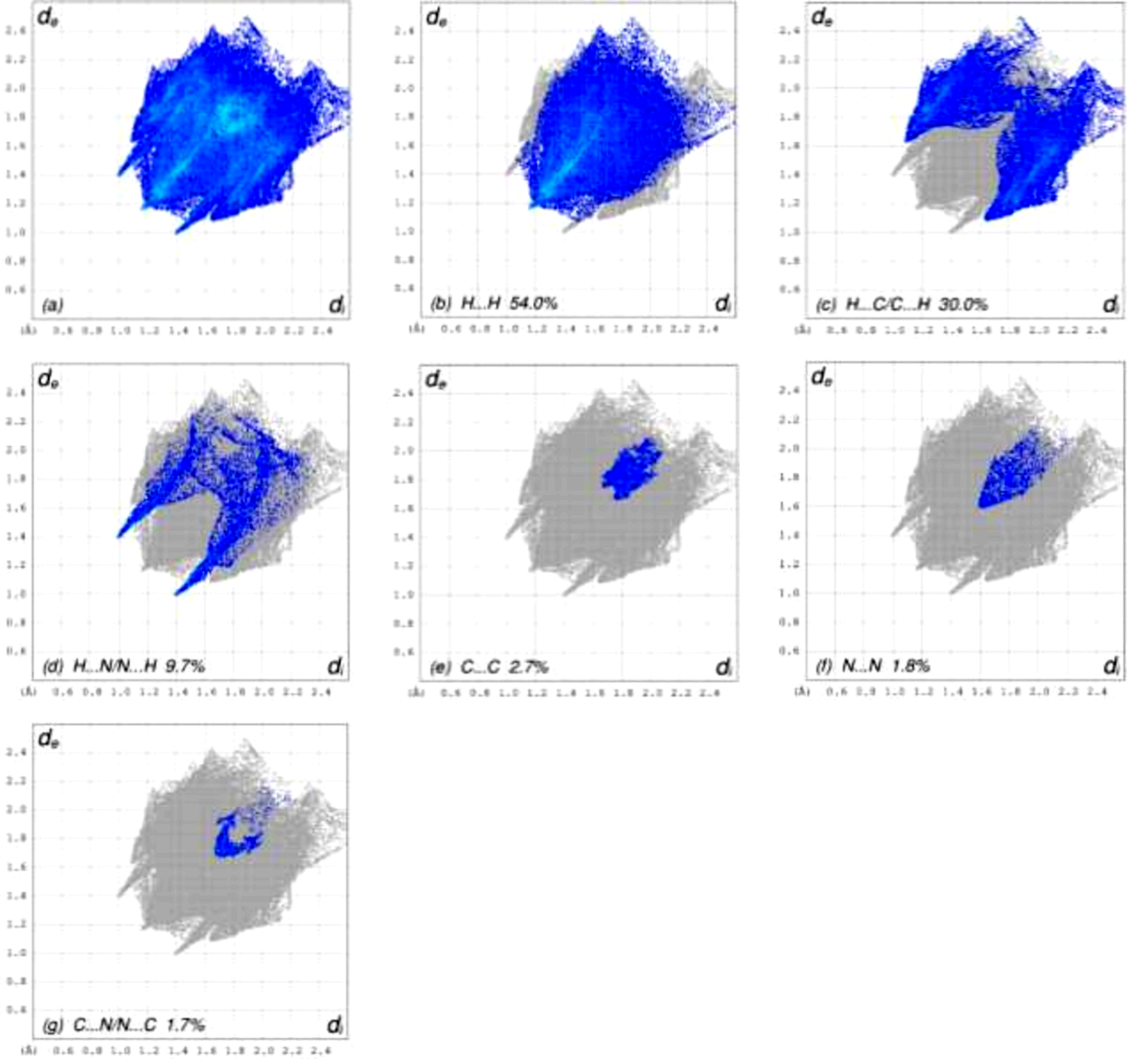
Two-dimensional fingerprint plots for **I**, showing (*a*) all inter­actions, and delineated into the different contact types (*b*)–(*g*).

Hirshfeld surface analyses for **I** and **II** {*catena*-poly[[(μ_2_-2,3-di­phenyl­pyrazine)­silver(I)] tetra­fluoro­borate nitro­methane solvate]; CSD refcode EQOYIS; Schultheiss *et al.*, 2003 ▸} were carried out using *CrystalExplorer* (Version 17.5; Spackman *et al.*, 2021 ▸; Spackman *et al.*, 2008 ▸; McKinnon *et al.*, 2007 ▸; Turner *et al.*, 2015 ▸). The Hirshfeld surface for **I** is shown in Fig. 3 ▸, where the bright-red spots correspond to the respective donors and acceptors noted above. The Hirshfeld surfaces for the two mol­ecules in BOHPOD are shown in Figs. S1(*a*) and S1(*b*) in the supporting information. The contact-type per­cen­tages from the two-dimensional fingerprint plot for **I** (Fig. 4 ▸) and **II** (Figs. S3) are listed in Table 2 ▸. These data show that the contact per­cen­tages are similar, with H⋯H and C⋯H/H⋯C dominating in each case.

## 5. Inter­action energy calculations and energy frameworks

**Figure 5:**
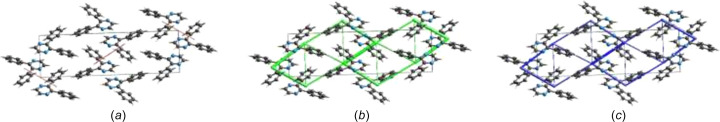
The energy frameworks for a cluster of mol­ecules of **I**, viewed down the *a*-axis direction, showing (*a*) electrostatic energy, (*b*) dispersion energy and (*c*) total energy diagrams. The cylindrical radius is proportional to the relative strength of the corresponding energies and they were adjusted to the same scale factor of 80 with a cut-off value of 5 kJ mol^−1^ within 2 × 2 × 2 unit cells.

The CE-B3LYP/6-31G(d,p) energy model available in *CrystalExplorer* was used to calculate the inter­molecular inter­action energies in **I**. The inter­action energies (in kJ mol^−1^) were calculated to be −7.6 (*E*_ele_), −2.4 (*E*_pol_), −40.4 (*E*_dis_), +27.0 (*E*_rep_) and −28.3 (*E*_tot_) for the C6—H6⋯N1^i^ hy­dro­gen-bond inter­action. Energy frameworks were constructed for *E*_ele_ (red cylinders), *E*_dis_ (green cylinders) and *E*_tot_ (blue cylinders) [Figs. 5 ▸(*a*), 5(*b*) and 5(*c*)], and their evaluation indicate that the stabilization is dominated by dispersion energy contributions in the crystal structure of **I**. A similar calculation for BOHPOD (Figs. S4 and S5) indicates that the stabilization is also dominated by the dispersion energy contributions.

## 6. Database survey

A survey of the Cambridge Structural Database (CSD, July 2025 update; Groom *et al.*, 2016 ▸) revealed several structures where 2,3-di­phenyl­pyrazine can act as a ligand, either *N*-bonded or *N*,*C*-bonded, or as an anion. They include refcode BOHPOD (Kitano *et al.*, 1983 ▸), **II** (EQOYIS; Schultheiss *et al.*, 2003 ▸), **III** (HABSAI; Hrovat *et al.*, 2020 ▸), **IV** (IFELOX; Zhu *et al.*, 2018 ▸), **V** (IQUJAJ; Shi *et al.*, 2025 ▸), **VI** (KAQHES; Luo *et al.*, 2017 ▸), **VII** (LEXDAW; Tian *et al.*, 2018 ▸), **VIII** (LEXDEA; Tian *et al.*, 2018 ▸), **IX** (LEXDIE; Tian *et al.*, 2018 ▸), **X** (LEXFEC; Tian *et al.*, 2018 ▸), **XI** (LEXFIG; Tian *et al.*, 2018 ▸), **XII** (REJLAW; Tian *et al.*, 2016 ▸), **XIII** (REJLIE; Tian *et al.*, 2016 ▸) and **XIV** (VIBXAF; Steel & Caygill, 1990 ▸). The dihedral angles between the central and pendant rings for these structures are given in the supporting information.

## 7. Synthesis and crystallization

Ethyl­enedi­amine (60.1 mg, 1.0 mmol) was added to a solution of benzil (210.2 mg, 1.0 mmol) in ethanol (50 ml). The reaction mixture was refluxed for 4 h, cooled to room tem­per­a­ture for precipitation and then filtered. Yellow crystals of **I** suitable for X-ray analysis were obtained by slow evaporation of an ethanol solution (yield 88%; m.p. 115–116 °C). Elemental analysis calculated (%) for C_16_H_12_N_2_: C 82.73, H 5.21, N 12.06; found: C 82.75, H 5.19, N 12.08. The report of Kitano *et al.* (1983 ▸) unfortunately does not mention the synthesis or (re)crystallization conditions for **II**.

## 8. Refinement

Crystal data, data collection and structure refinement details are summarized in Table 3 ▸. The H-atom positions were calculated geometrically (C—H = 0.95 Å) and refined using a riding model by applying the constraint *U*_iso_(H) = 1.2*U*_eq_(C).

## Supplementary Material

Crystal structure: contains datablock(s) I, global. DOI: 10.1107/S2056989026000460/hb8177sup1.cif

Structure factors: contains datablock(s) I. DOI: 10.1107/S2056989026000460/hb8177Isup2.hkl

Additional figures. DOI: 10.1107/S2056989026000460/hb8177sup3.pdf

Supporting information file. DOI: 10.1107/S2056989026000460/hb8177Isup4.cml

CCDC reference: 2524118

Additional supporting information:  crystallographic information; 3D view; checkCIF report

## Figures and Tables

**Table 1:** Hydrogen-bond geometry (Å, °) *Cg*2 and *Cg*3 are the centroids of the C7–C12 and C13–C18 rings, respectively.

*D*—H⋯*A*	*D*—H	H⋯*A*	*D*⋯*A*	*D*—H⋯*A*
C6—H6⋯N1^i^	0.95	2.52	3.3740 (15)	149
C12—H12⋯*Cg*3^ii^	0.95	2.97	3.7734 (12)	143
C16—H16⋯*Cg*2^iii^	0.95	2.99	3.8974 (13)	159

Symmetry codes: (i) 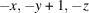; (ii) 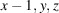; (iii) 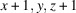.

**Table 2:** Comparison of the contact-type per­cen­tages for title com­pound **I** and BOHPOD, where the asymmetric unit contains two crystallographically independent mol­ecules, *A* and *B*

Contact	**I**	BOHPOD (*A*)	BOHPOD (*B*)
H⋯H	54.0	47.7	55.1
H⋯C/C⋯H	30.0	34.1	31.6
H⋯N/N⋯H	9.7	14.7	12.2
C⋯C	2.7	1.5	0.8
N⋯N	1.8	0.1	0.0
C⋯N/N⋯C	1.7	1.9	0.3

**Table 3:** Experimental details

Crystal data	
Chemical formula	C_16_H_12_N_2_
*M*_r_	232.28
Crystal system, space group	Monoclinic, *P*2_1_/*c*
Temperature (K)	173
*a*, *b*, *c* (Å)	6.2885 (2), 25.4882 (9), 7.5554 (3)
β (°)	94.403 (4)
*V* (Å^3^)	1207.43 (8)
*Z*	4
Radiation type	Mo *K*α
μ (mm^−1^)	0.08
Crystal size (mm)	0.22 × 0.09 × 0.02

Data collection	
Diffractometer	Rigaku XtaLAB P200K
Absorption correction	Multi-scan (*CrysAlis PRO*; Rigaku OD, 2024 ▸)
*T*_min_, *T*_max_	0.812, 1.000
No. of measured, independent and observed [*I* > 2σ(*I*)] reflections	26279, 3011, 2387
*R*_int_	0.031
(sin θ/λ)_max_ (Å^−1^)	0.696

Refinement	
*R*[*F*^2^ > 2σ(*F*^2^)], *wR*(*F*^2^), *S*	0.039, 0.097, 1.03
No. of reflections	3011
No. of parameters	163
H-atom treatment	H-atom parameters constrained
Δρ_max_, Δρ_min_ (e Å^−3^)	0.21, −0.17

Computer programs: *CrysAlis PRO* (Rigaku OD, 2024 ▸), *SHELXT2018* (Sheldrick, 2015*a* ▸), *SHELXL2019* (Sheldrick, 2015*b* ▸) and *OLEX2* (Dolomanov *et al.*, 2009 ▸).

## supplementary crystallographic information

### 2,3-Diphenylpyrazine Crystal data

**Table:**

C_16_H_12_N_2_	*F*(000) = 488
*M**_r_* = 232.28	*D*_x_ = 1.278 Mg m^−^^3^
Monoclinic, *P*2_1_/*c*	Mo *K*α radiation, λ = 0.71073 Å
*a* = 6.2885 (2) Å	Cell parameters from 7919 reflections
*b* = 25.4882 (9) Å	θ = 2.7–29.0°
*c* = 7.5554 (3) Å	µ = 0.08 mm^−^^1^
β = 94.403 (4)°	*T* = 173 K
*V* = 1207.43 (8) Å^3^	Prism, colourless
*Z* = 4	0.22 × 0.09 × 0.02 mm

### 2,3-Diphenylpyrazine Data collection

**Table:**

Rigaku XtaLAB P200K diffractometer	3011 independent reflections
Radiation source: Rotating Anode, Rigaku FR-X	2387 reflections with *I* > 2σ(*I*)
Rigaku Osmic Confocal Optical System monochromator	*R*_int_ = 0.031
Detector resolution: 5.8140 pixels mm^-1^	θ_max_ = 29.6°, θ_min_ = 2.8°
shutterless scans	*h* = −8→8
Absorption correction: multi-scan (CrysAlis PRO; Rigaku OD, 2024)	*k* = −33→35
*T*_min_ = 0.812, *T*_max_ = 1.000	*l* = −7→10
26279 measured reflections	

### 2,3-Diphenylpyrazine Refinement

**Table:**

Refinement on *F*^2^	Primary atom site location: dual
Least-squares matrix: full	Hydrogen site location: inferred from neighbouring sites
*R*[*F*^2^ > 2σ(*F*^2^)] = 0.039	H-atom parameters constrained
*wR*(*F*^2^) = 0.097	*w* = 1/[σ^2^(*F*_o_^2^) + (0.0426*P*)^2^ + 0.2327*P*] where *P* = (*F*_o_^2^ + 2*F*_c_^2^)/3
*S* = 1.03	(Δ/σ)_max_ < 0.001
3011 reflections	Δρ_max_ = 0.21 e Å^−^^3^
163 parameters	Δρ_min_ = −0.17 e Å^−^^3^
0 restraints	

### 2,3-Diphenylpyrazine Special details

**Table:**

Geometry. All esds (except the esd in the dihedral angle between two l.s. planes) are estimated using the full covariance matrix. The cell esds are taken into account individually in the estimation of esds in distances, angles and torsion angles; correlations between esds in cell parameters are only used when they are defined by crystal symmetry. An approximate (isotropic) treatment of cell esds is used for estimating esds involving l.s. planes.

### 2,3-Diphenylpyrazine Fractional atomic coordinates and isotropic or equivalent isotropic displacement parameters (Å^2^)

**Table:**

	*x*	*y*	*z*	*U*_iso_*/*U*_eq_	
N1	0.22049 (15)	0.45593 (4)	0.09624 (13)	0.0358 (2)	
N4	0.56788 (15)	0.49704 (3)	0.29880 (12)	0.0332 (2)	
C2	0.37217 (16)	0.42424 (4)	0.17179 (14)	0.0281 (2)	
C3	0.54118 (16)	0.44465 (4)	0.28378 (13)	0.0274 (2)	
C5	0.42219 (19)	0.52761 (4)	0.21441 (14)	0.0359 (3)	
H5	0.441288	0.564571	0.218955	0.043*	
C6	0.24486 (19)	0.50752 (4)	0.12082 (16)	0.0380 (3)	
H6	0.137428	0.530775	0.072621	0.046*	
C7	0.34590 (17)	0.36787 (4)	0.12216 (13)	0.0293 (2)	
C8	0.50968 (18)	0.34009 (4)	0.05075 (15)	0.0360 (3)	
H8	0.643471	0.356536	0.039207	0.043*	
C9	0.4783 (2)	0.28844 (5)	−0.00368 (16)	0.0430 (3)	
H9	0.590011	0.269856	−0.053831	0.052*	
C10	0.2851 (2)	0.26396 (4)	0.01483 (16)	0.0444 (3)	
H10	0.264447	0.228505	−0.021280	0.053*	
C11	0.1226 (2)	0.29118 (5)	0.08586 (17)	0.0441 (3)	
H11	−0.009944	0.274330	0.099417	0.053*	
C12	0.15138 (18)	0.34301 (4)	0.13762 (15)	0.0359 (3)	
H12	0.037361	0.361700	0.184020	0.043*	
C13	0.69606 (16)	0.41323 (4)	0.39840 (13)	0.0275 (2)	
C14	0.89935 (17)	0.43358 (4)	0.44188 (15)	0.0331 (2)	
H14	0.940374	0.465469	0.388999	0.040*	
C15	1.04192 (18)	0.40806 (5)	0.56072 (17)	0.0411 (3)	
H15	1.179558	0.422512	0.589169	0.049*	
C16	0.9848 (2)	0.36156 (5)	0.63825 (17)	0.0433 (3)	
H16	1.082815	0.343962	0.719784	0.052*	
C17	0.7840 (2)	0.34079 (4)	0.59647 (16)	0.0405 (3)	
H17	0.744329	0.308866	0.649905	0.049*	
C18	0.63994 (18)	0.36615 (4)	0.47724 (15)	0.0333 (2)	
H18	0.502664	0.351456	0.449107	0.040*	

### 2,3-Diphenylpyrazine Atomic displacement parameters (Å^2^)

**Table:**

	*U*^11^	*U*^22^	*U*^33^	*U*^12^	*U*^13^	*U*^23^
N1	0.0348 (5)	0.0320 (5)	0.0400 (5)	0.0054 (4)	−0.0009 (4)	0.0013 (4)
N4	0.0421 (5)	0.0245 (4)	0.0328 (5)	−0.0017 (4)	0.0023 (4)	0.0005 (3)
C2	0.0291 (5)	0.0261 (5)	0.0291 (5)	0.0015 (4)	0.0031 (4)	0.0009 (4)
C3	0.0309 (5)	0.0243 (5)	0.0274 (5)	−0.0001 (4)	0.0050 (4)	0.0007 (4)
C5	0.0501 (7)	0.0232 (5)	0.0347 (6)	0.0040 (4)	0.0054 (5)	0.0012 (4)
C6	0.0423 (6)	0.0312 (6)	0.0404 (6)	0.0101 (5)	0.0029 (5)	0.0037 (5)
C7	0.0332 (5)	0.0270 (5)	0.0267 (5)	0.0013 (4)	−0.0045 (4)	−0.0001 (4)
C8	0.0378 (6)	0.0324 (6)	0.0375 (6)	0.0024 (5)	0.0010 (5)	−0.0015 (5)
C9	0.0555 (7)	0.0334 (6)	0.0398 (7)	0.0110 (5)	0.0012 (6)	−0.0041 (5)
C10	0.0628 (8)	0.0265 (5)	0.0418 (7)	−0.0010 (5)	−0.0100 (6)	−0.0042 (5)
C11	0.0454 (7)	0.0368 (6)	0.0485 (7)	−0.0098 (5)	−0.0073 (6)	−0.0015 (5)
C12	0.0344 (6)	0.0340 (6)	0.0382 (6)	−0.0014 (4)	−0.0036 (5)	−0.0031 (5)
C13	0.0310 (5)	0.0251 (5)	0.0264 (5)	0.0004 (4)	0.0010 (4)	−0.0035 (4)
C14	0.0334 (5)	0.0291 (5)	0.0368 (6)	−0.0022 (4)	0.0032 (5)	−0.0039 (4)
C15	0.0328 (6)	0.0433 (7)	0.0456 (7)	0.0012 (5)	−0.0062 (5)	−0.0091 (5)
C16	0.0488 (7)	0.0408 (6)	0.0382 (6)	0.0118 (5)	−0.0108 (5)	−0.0029 (5)
C17	0.0549 (7)	0.0286 (5)	0.0370 (6)	0.0036 (5)	−0.0024 (5)	0.0035 (5)
C18	0.0374 (6)	0.0275 (5)	0.0344 (6)	−0.0028 (4)	−0.0008 (5)	0.0001 (4)

### 2,3-Diphenylpyrazine Geometric parameters (Å, º)

**Table:**

N1—C2	1.3434 (13)	C10—H10	0.9500
N1—C6	1.3352 (15)	C10—C11	1.3776 (18)
N4—C3	1.3495 (13)	C11—H11	0.9500
N4—C5	1.3280 (14)	C11—C12	1.3856 (16)
C2—C3	1.4065 (14)	C12—H12	0.9500
C2—C7	1.4908 (14)	C13—C14	1.3953 (14)
C3—C13	1.4867 (14)	C13—C18	1.3970 (15)
C5—H5	0.9500	C14—H14	0.9500
C5—C6	1.3728 (17)	C14—C15	1.3819 (16)
C6—H6	0.9500	C15—H15	0.9500
C7—C8	1.3923 (15)	C15—C16	1.3817 (18)
C7—C12	1.3905 (15)	C16—H16	0.9500
C8—H8	0.9500	C16—C17	1.3832 (18)
C8—C9	1.3888 (16)	C17—H17	0.9500
C9—H9	0.9500	C17—C18	1.3873 (16)
C9—C10	1.3824 (18)	C18—H18	0.9500
			
C6—N1—C2	117.58 (10)	C11—C10—H10	120.1
C5—N4—C3	117.61 (9)	C10—C11—H11	119.8
N1—C2—C3	120.82 (9)	C10—C11—C12	120.35 (11)
N1—C2—C7	114.29 (9)	C12—C11—H11	119.8
C3—C2—C7	124.88 (9)	C7—C12—H12	119.8
N4—C3—C2	120.00 (9)	C11—C12—C7	120.50 (11)
N4—C3—C13	114.35 (9)	C11—C12—H12	119.8
C2—C3—C13	125.60 (9)	C14—C13—C3	118.97 (9)
N4—C5—H5	118.9	C14—C13—C18	118.41 (10)
N4—C5—C6	122.12 (10)	C18—C13—C3	122.37 (9)
C6—C5—H5	118.9	C13—C14—H14	119.5
N1—C6—C5	121.26 (10)	C15—C14—C13	120.99 (10)
N1—C6—H6	119.4	C15—C14—H14	119.5
C5—C6—H6	119.4	C14—C15—H15	119.9
C8—C7—C2	121.11 (10)	C16—C15—C14	120.16 (11)
C12—C7—C2	119.95 (9)	C16—C15—H15	119.9
C12—C7—C8	118.85 (10)	C15—C16—H16	120.2
C7—C8—H8	119.9	C15—C16—C17	119.62 (11)
C9—C8—C7	120.28 (11)	C17—C16—H16	120.2
C9—C8—H8	119.9	C16—C17—H17	119.7
C8—C9—H9	119.9	C16—C17—C18	120.59 (11)
C10—C9—C8	120.26 (11)	C18—C17—H17	119.7
C10—C9—H9	119.9	C13—C18—H18	119.9
C9—C10—H10	120.1	C17—C18—C13	120.23 (10)
C11—C10—C9	119.74 (11)	C17—C18—H18	119.9
			
N1—C2—C3—N4	−8.41 (15)	C5—N4—C3—C13	−173.21 (9)
N1—C2—C3—C13	168.90 (10)	C6—N1—C2—C3	4.82 (15)
N1—C2—C7—C8	125.29 (11)	C6—N1—C2—C7	−173.66 (10)
N1—C2—C7—C12	−51.08 (13)	C7—C2—C3—N4	169.91 (9)
N4—C3—C13—C14	−29.59 (13)	C7—C2—C3—C13	−12.78 (16)
N4—C3—C13—C18	144.59 (10)	C7—C8—C9—C10	−0.88 (18)
N4—C5—C6—N1	−6.40 (18)	C8—C7—C12—C11	1.19 (16)
C2—N1—C6—C5	2.32 (16)	C8—C9—C10—C11	0.69 (18)
C2—C3—C13—C14	152.97 (10)	C9—C10—C11—C12	0.44 (18)
C2—C3—C13—C18	−32.85 (16)	C10—C11—C12—C7	−1.39 (18)
C2—C7—C8—C9	−176.47 (10)	C12—C7—C8—C9	−0.06 (16)
C2—C7—C12—C11	177.64 (10)	C13—C14—C15—C16	0.21 (18)
C3—N4—C5—C6	2.75 (16)	C14—C13—C18—C17	0.35 (16)
C3—C2—C7—C8	−53.13 (15)	C14—C15—C16—C17	−0.12 (18)
C3—C2—C7—C12	130.50 (11)	C15—C16—C17—C18	0.15 (18)
C3—C13—C14—C15	174.09 (10)	C16—C17—C18—C13	−0.27 (18)
C3—C13—C18—C17	−173.86 (10)	C18—C13—C14—C15	−0.32 (16)
C5—N4—C3—C2	4.39 (15)		

### 2,3-Diphenylpyrazine Hydrogen-bond geometry (Å, º)

Cg2 and Cg3 are the centroids of the C7–C12 and C13–C18 rings, respectively.

**Table:**

*D*—H···*A*	*D*—H	H···*A*	*D*···*A*	*D*—H···*A*
C6—H6···N1^i^	0.95	2.52	3.3740 (15)	149
C12—H12···*Cg*3^ii^	0.95	2.97	3.7734 (12)	143
C16—H16···*Cg*2^iii^	0.95	2.99	3.8974 (13)	159

Symmetry codes: (i) −*x*, −*y*+1, −*z*; (ii) *x*−1, *y*, *z*; (iii) *x*+1, *y*, *z*+1.

### Comparison of the dihedral angles (°) between the central and pendant rings for some structures

**Table:**

Compound	α	α	α	α	α	α	α	α
I	53.12 (3)	33.28 (3)	–	–	–	–	–	–
III	56.56	57.73	–	–	–	–	–	–
V	13.58	54.30	–	–	–	–	–	–
VI	24.09	22.75	–	–	–	–	–	–
VII	62.45	68.83	43.77	55.12	42.85	51.17	57.23	51.87
VIII	56.47	30.59	56.33	72.22	44.84	66.42	52.59	53.31
IX	53.25	50.38	–	–	–	–	–	–
X	66.72	65.00	61.37	54.57	57.76	46.71	52.78	64.28
XI	57.69	31.21	56.67	54.09	74.66	23.83	–	–
