# Supplementary material for: A second monoclinic polymorph of 2,3-di­phenyl­pyrazine
Source: Acta Crystallogr E Crystallogr Commun. 2026 Jan 23;82(Pt 2):194–7. doi: 10.1107/S2056989026000460 (PMC12874241; doi:10.1107/S2056989026000460)
Supplement: Supplementary file 3 [file e-82-00194-sup3.pdf]

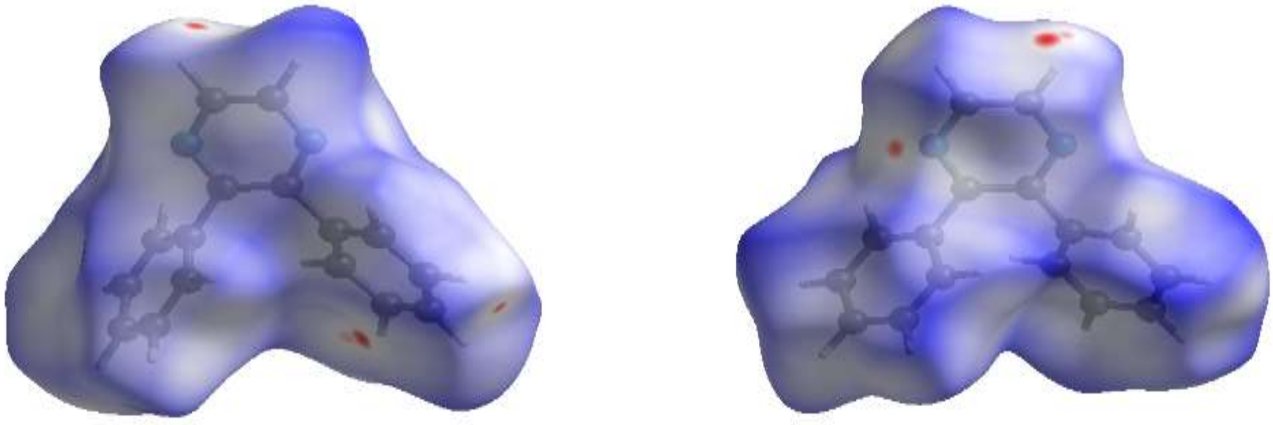

(a) (b)

Figure S1. Views of the three-dimensional Hirshfeld surfaces of BOHPOD plotted over d_{norm} (a) molecule I and (b) molecule II in the ranges of -0.0574 to 1.4999 a.u. (for molecule I) and -0.0595 to 1.3454 a.u. (for molecule II).

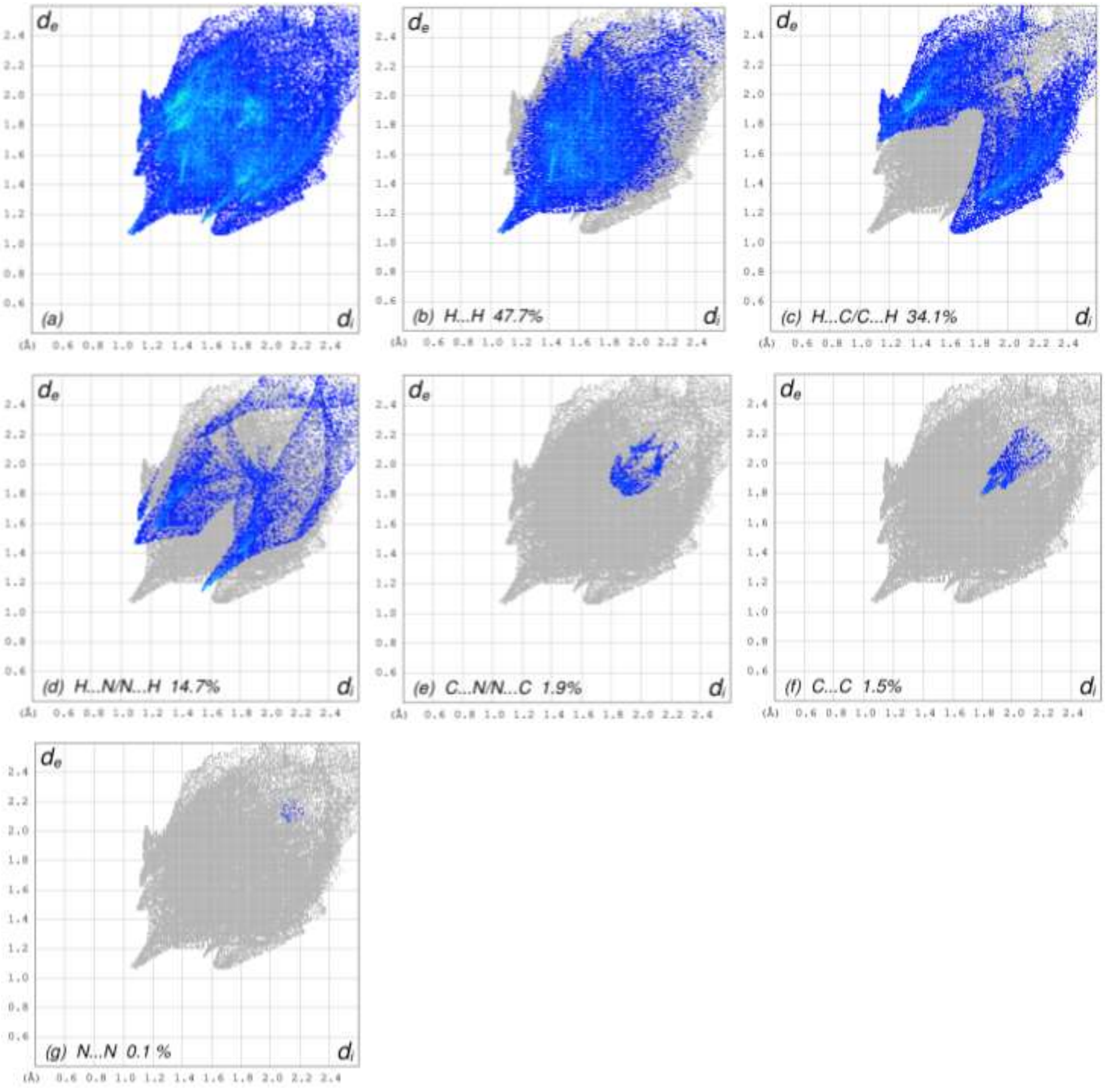

Figure S2. The full two-dimensional fingerprint plots for molecule **I**, showing (a) all interactions, and delineated into (b) H ... H, (c) H ... C/C ... H, (d) H ... N/N ... H, (e) C ... N/N ... C, (f) C ... C and (g) N ... N interactions. The d_i and d_e values are the closest internal and external distances (in Å) from given points on the Hirshfeld surface contacts.

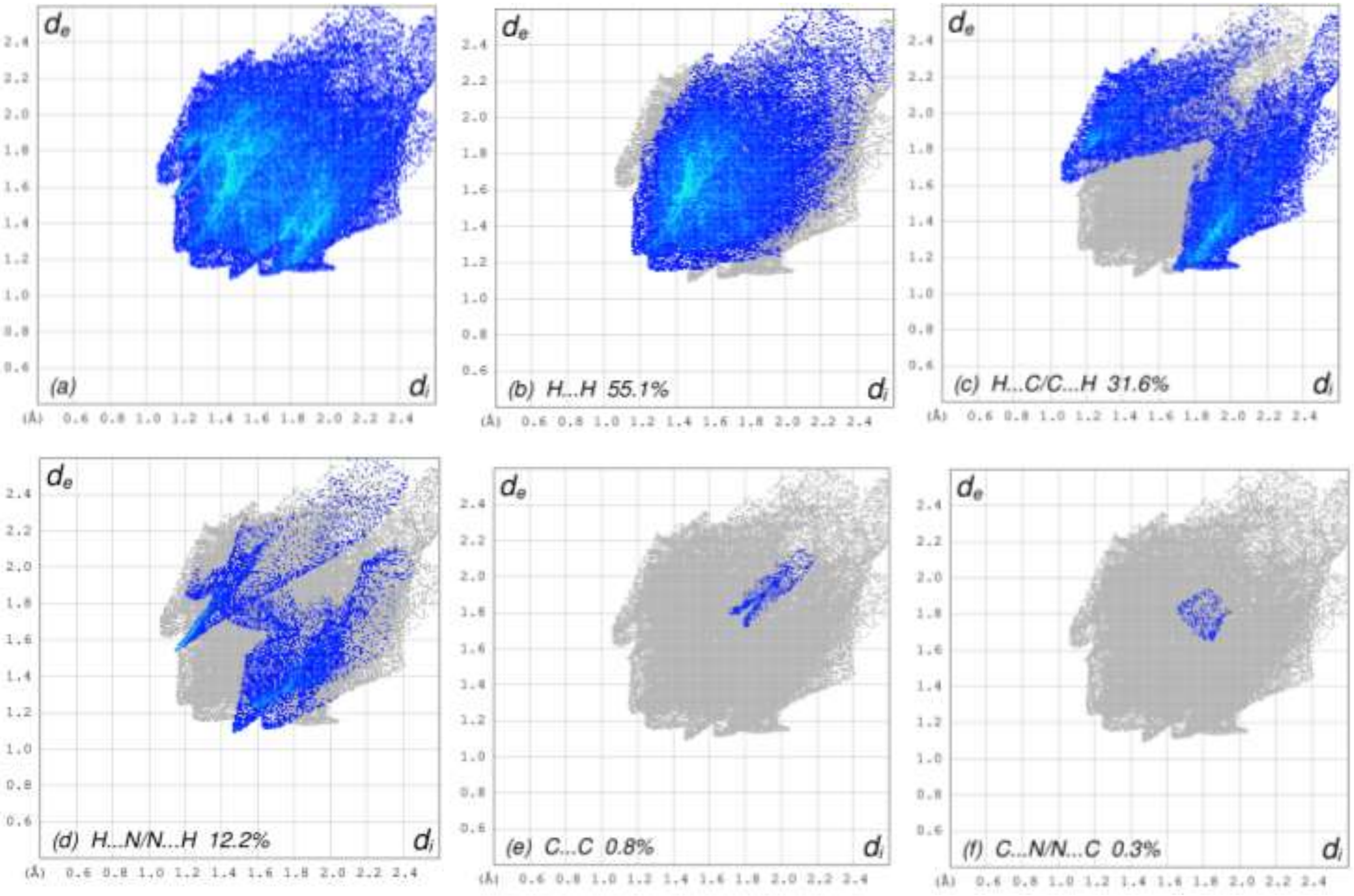

Figure S3. The full two-dimensional fingerprint plots for molecule **I**, showing (a) all interactions, and delineated into (b) H ... H, (c) H ... C/C ... H, (d) H ... N/N ... H, (e) C ... C and (f) C ... N/N ... C interactions. The d_i and d_e values are the closest internal and external distances (in Å) from given points on the Hirshfeld surface contacts.

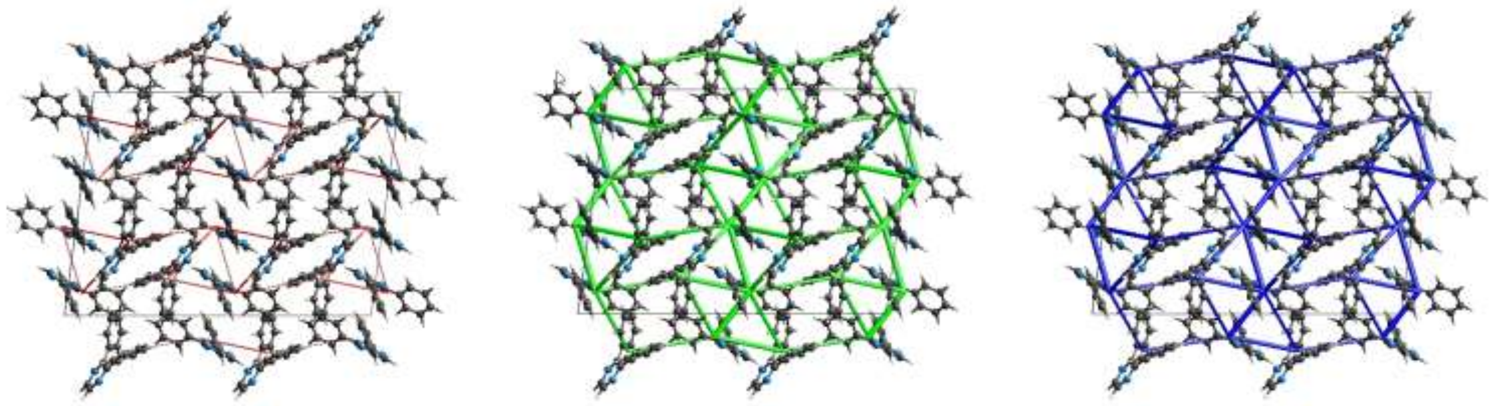

(a) (b) (c)

Figure S4. The energy frameworks for a cluster of molecules of molecule **I** viewed down the *b*-axis direction showing the (a) electrostatic energy, (b) dispersion energy and (c) total energy diagrams. The cylindirical radius is proportional to the relative strength of the corresponding energies and they were adjusted to the same scale factor of 80 with cut-off value of 5 kJ mol^{-1} within 2 X 2 X 2 unit cells.

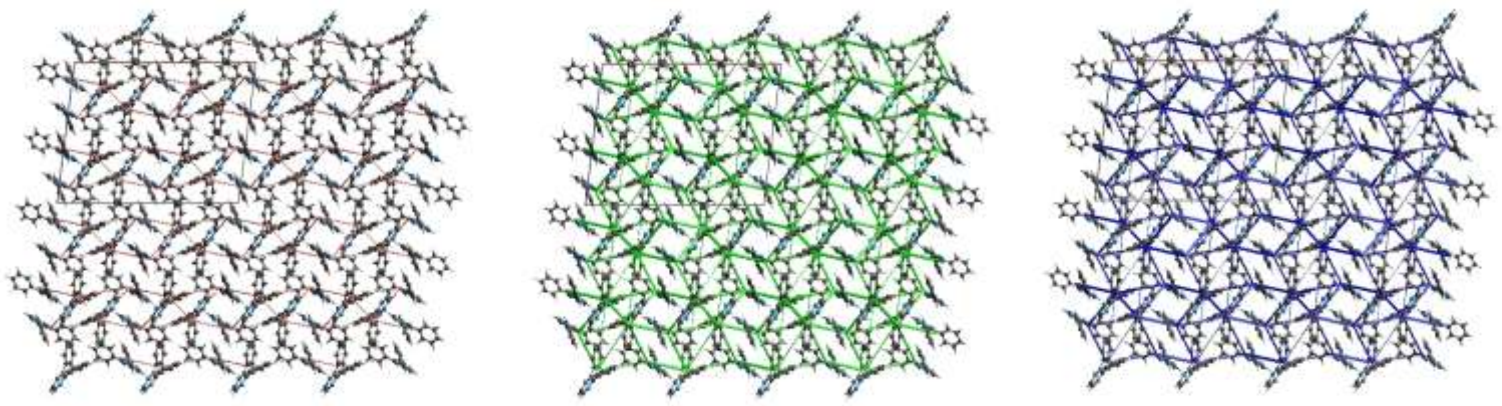

(a) (b) (c)

Figure S5. The energy frameworks for a cluster of molecules of molecule **II** viewed down the *b*-axis direction showing the (a) electrostatic energy, (b) dispersion energy and (c) total energy diagrams. The cylindirical radius is proportional to the relative strength of the corresponding energies and they were adjusted to the same scale factor of 80 with cut-off value of 5 kJ mol^{-1} within 2 X 2 X 2 unit cells.
